# Reduction of downtime and improvement of the utility of a conventional radiotherapy simulator

**DOI:** 10.1120/jacmp.v4i4.2504

**Published:** 2003-09-01

**Authors:** S. P. Sawchuk, K. Knibutat, L. Moriarity

**Affiliations:** ^1^ Department of Oncology University of Calgary 2500 University Dr. NW Calgary Alberta T2N 1N4 Canada; ^2^ Department of Medical Physics Tom Baker Cancer Centre 1331 29 St. NW Calgary Alberta T2N 1N4 Canada; ^3^ Department of Medical Physics Tom Baker Cancer Centre 1331 29 St. NW Calgary Alberta T2N 1N4 Canada

**Keywords:** conventional simulator, downtime, field light, optical distance indicator, reticule

## Abstract

The modifications made to a radiotherapy simulator that improve its functionality and minimize its downtime are described. Functionality was enhanced with some simple effective improvements. Decreasing the frequency and time spent on repairs reduced downtime. These “in‐house” improvements were made to a Varian Ximatron simulator at the Tom Baker Cancer Center. Now there is easier access to the field light and optical distance indicator (ODI). Projections of the field light and ODI are much brighter and ODI adjustment is more reproducible. A crosshair was etched onto the clear *x*‐ray exit window to optically overlap with the crosshair of the reticule. Now, the time required to replace a field light is up to seven times faster and the time to realign a reticule is reduced to the order of three times faster. Only one quality control (QC) check is required after these adjustments. Removal of excess heat within the gantry head has eliminated two major problems: (A) the metal rod supporting the mirror assembly for the field light no longer expands and contracts causing a misalignment of the field light projection, and (B) the damage to the bearing in the cooling fan assembly has been eliminated. The simulator is now more functional and these improvements have reduced downtime significantly, making repairs and adjustments much more convenient and efficient.

PACS number(s): 87.53.Vb, 87.53.Xd

## INTRODUCTION

Conventional radiotherapy simulators are still the workhorses for treatment simulation even with the popularity of CT‐simulators and virtual simulation. Almost all treatment facilities use conventional simulation for tumor localization and treatment simulation for many treatment sites. They are much less expensive and simpler and cheaper to repair and maintain than the more complicated CT‐simulators.^1^ They can accommodate patients with larger encompassing treatment positions more readily than most CT‐simulators. They are definitely more convenient to use for simple treatment cases, can capture real‐time patient movement such as breathing motion, and can also be used as a convenient backup to CT‐simulators. Hence, it is imperative to keep these important machines operating with minimal downtime.

Some conventional simulator designs do not allow convenient or timely repair and replacement of components within the gantry simulator head. The compactness of all of the various systems makes it difficult to gain access to the affected component and not adversely affect other nearby components unintentionally. Thus, seemingly simple routine servicing that should be quick to complete are made difficult and untimely.

The replacement of burnt out light bulbs for the field light and optical distance indicator (ODI) assemblies require immediate replacement usually during a busy clinical day. On the average replacement occurs once or twice a year and there have been years where light bulb replacement occurred up to four times. The proximity of these lights is so close to other crucial components that any tampering with them may adversely affect the others. Again what should be simple tasks are manifest into inordinate amounts of time during the useful clinical day causing an increase in downtime that should be preventable or reducible.

**Figure 1:**
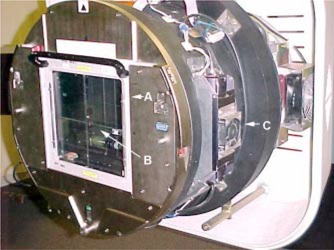
(Color) Simulator gantry head with covers removed. A, reticule; B, exit window, and C, modified field light/ODI/ cooling fan assembly.

All improvements have been made to the components in the head of the simulator gantry with the intent of making convenient and more efficient adjustments and major savings in downtime. Before modifications downtime from these components ranged from 5.5 to 22.5 h per year, and after the improvements downtime was reduced to the range of 3.5 to 5.5 h. The time to plan trouble shoot, and implement the modifications took approximately one week of nonclinical time. Duplicate original equipment manufacturer's (OEM) components were modified externally allowing continued clinical use of the simulator. Final implementation of the completed modules was efficient and also performed outside of clinical time (see Table II). The importance of the field light, ODI, and reticule alignment (see Fig. 1) in the simulator is crucial for patient positioning accuracy during patient treatment.^2^

Seven major improvements have been made to deal with the following problems:

(i) Gaining access to the affected device in a timely manner within a tightly compacted and confined space. Many components are within very close proximity to one another within the simulator head. In fact, some systems block access to others and some systems cannot be handled without simultaneously touching and adversely affecting another.
(ii) Heat buildup caused by the burning bulbs of the field light and ODI. This heating and eventual cooling causes the metal rod supporting the mirror to expand and contract. The mirror, which is then a moving target, changes the optical projection of the reticule crosshair causing a perceived misalignment. In the cooled state, this misalignment disappears.
(iii) The cooling fan bearing wears down with latent heat originating from hot light bulbs causing premature failure.
(iv) Alignment mechanism for ODI lacks reproducible control using only one screw to control tilt. Thus, trial and error is used in positioning.
(v) Projections of the field light and the ODI are dim enough that they are difficult to read on the patient.
(vi) Damaged or loose reticules have been time consuming to realign.
(vii) Quality control checks after any of the above mentioned repairs have been very time consuming and may have to be repeated in an iterative repair procedure causing further delay to clinical service. Reducing repetition of these or eliminating them altogether is desirable in reducing downtime.

## METHODS

### Adjustments

**Figure 2:**
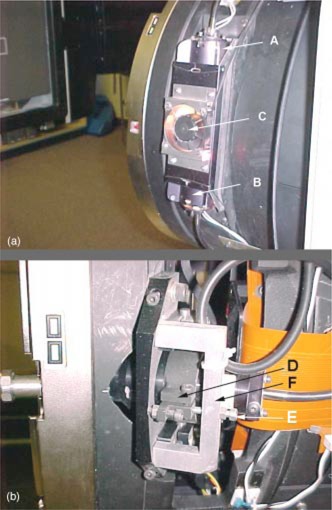
(Color) Side of simulator gantry head with covers removed. (a) A, ODI, B, Field Light, C, cooling fan, (b) D, ODI assembly, E, ODI locking bracket, F, ODI clamp.

The lamp assembly was modified with fiber optics (Varian Medical Systems. “Ximatron C‐Series.” Product Manual. Section 10: Drawings and Diagrams. TM59610000 Issue 9. Sept. 1995. Collimator: ODI Light Box Assembly. Drawing No. TM55737000) to make the manipulation of the assembly more convenient and timely. It was relocated to share the housing (Varian Medical Systems. “Ximatron C‐Series.” Product Manual. Section 10: Drawings and Diagrams. TM59610000 Issue 9. Sept. 1995. Collimator: Collimator Assembly. Drawing No. TM56088000) with the ODI and the cooling fan that is shown in Fig. 2. The fiber optic cable simply attaches to the housing with a clamping mechanism.

**Figure 3:**
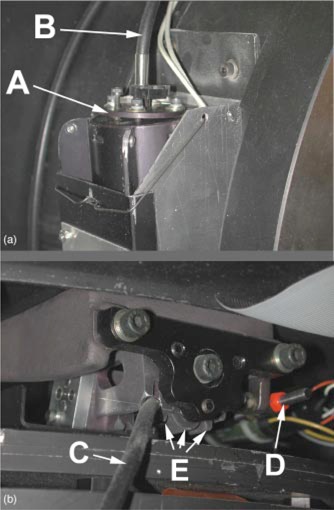
(Color) Lamp assembly showing (a) A tilt mechanism and B fiber optic, (b) C fiber optic cable for field light, and D mirror supporting rod, and E metal cooling fins.

A special tilting mechanism was added to the fiber optic inputs for the field light and ODI (Varian Medical Systems. “Ximatron C‐Series.” Product Manual. Section 10: Drawings and Diagrams. TM59610000 Issue 9. Sept. 1995. ODI Set‐Up. Drawing No. TM55441000) shown in Fig. 3. The respective bulbs can now be fixed into a more optimal optical position. Special calibration procedures were not required following these modifications other than radiation/field light coincidence and cross‐hair position checks.

The ODI mechanism for alignment was controlled from a single screw or pivot (Varian Oncology Systems. “Clinac 2100C/D.” Systems Manual. Section “Technical Support Documents.” Apr. 1990. Lamp Removal and Replacement.) It was modified to allow movements using two independently placed screws. This allows a push‐pull dynamic facilitating more reproducible movements for alignment. Figure 2 shows the new bracket and locking nut added for stability. The cooling fan near the ODI assembly would only run when the ODI light was switched on. It has been adjusted to run continuously. The clear acrylic exit window (Fig. 1) has been etched with a crosshair that aligns optically with the reticule crosshair. Table I summarizes the status of components before and after the simulator modifications.

**Table I:** Highlights of component status before and after simulator modifications.

	Component	Status	Comments
Before modifications	Field light	Separate housing.	
		No cooling fan and closed environment.	Heat generated by this light was not removed.
		Dim light intensity.	Shadow from apex of lamp in‐line with input.
		Not fiber optic.	Adjustments or replacements greatly influence reticule cross‐hair optics.
		Tightly packed.	
	ODI	Separate housing. Close to cooling fan.	Fan runs when light switched on only. Heat is trapped.
		1 screw to control tilt alignment.	Movements with this mechanism are trial and error.
		Fiber optic existing. Dim light intensity.	Shadow from apex of lamp in‐line with input.
	Cooling fan	Switched on only when ODI light on.	Bearing becomes heat stressed causing premature failure.
	Acrylic exit window Field light	Clear.	
After modifications		Shared housing with ODI and cooling fan. Light intensity greatly increased.	Moved close to fan for quick and easy cooling. Tilt feature allows bulb to be fixed to capture brightest area of reflector.
		Fiber optic.	Adjustments or replacements independent of cross‐hair optics. More easily accessible.
	ODI	Shared housing with field light and cooling fan.	Fan remains close but always on.
		2 screws control alignment	Movements and adjustments are more predictable.
		Light intensity greatly increased.	Tilt feature allows bulb to be fixed to capture brightest area of reflector.
	Cooling fan Acrylic exit window	Running continuously. Optical crosshair is etched on which aligns with reticule.	Increases lifetime of fan. Accurate starting point for repair or replacement.

## RESULTS AND DISCUSSION

All of the improvements have produced a reduction of downtime and more convenient repairs and maintenance (see Table II). The components requiring the most frequent servicing, namely the field light and ODI, were improved significantly. The modified design facilitates easier access to these components while minimizing or eliminating the potential for adverse effects to other components nearby. This increases efficiency by eliminating needless adjustments and the accompanying QC checks.3 Replacing the light bulb for the field light for example will no longer affect the bulb positioning since it is fixed within the new housing and this eliminates the lengthy amount of time that was required previously to reposition the bulb. Now the QC afterwards is done only once for the affected component only.

The heat that builds up from the field light and the ODI light was concentrated and made the assembly too hot to touch. The conduction of this heat caused the expansion of the mounting rod (see Fig. 3) that supports the mirror, making it move out of position. This in turn caused the projection of the crosshair to deviate from the collimator rotation axis. Thus, the field light could not be adjusted properly until the rod had cooled putting the mirror back into its original position. Using the previous design and waiting extra time for the rod to cool, which could take up to 30 min during an already lengthy and inconvenient field light adjustment process could increase frustration dramatically. Placing the field light in the same housing as the ODI and the cooling fan and keeping the fan running continuously allow immediate convection of the heat and total elimination of this problem. Removal of this heat has also increased the lifetime of the cooling fan since the bearing no longer fails prematurely.

**Table II:** Expectation of efficiency of component adjustment and replacement before and after simulator modifications.

Function	Time before modifications (h)	Time after modifications (h)	Time Saved (h)
Field light bulb replacing/repair	2–4	0.5	1.5–3.5
Cool down waiting time as part of	0.5	0.0	0.5
replacing field light bulb			
ODI bulb adjustment/repair	0.5–1	0.5	0—0.5
Reticule adjustment or replacement	1–4	1.0	0—3.0
QC time (0.5 hrs/check) # checks	1–5	1	0—2.0
Brightness assessment	Dim with difficulty seeing projections on patient	Brighter and no difficulty seeing projection on patient	Greatly improved
Planning, trouble shooting, and implementation of all improvements Estimate of implementation only of all improvements	1 week nonclinical time. Performed in parallel with functioning simulator 2–3 days

The tilting mechanism for the field light and ODI is a very simple but significant improvement. This feature locks the corresponding light bulb into a position eliminating the fiber optic input falling on the optical shadow created by the apex of the bulb. A significant increase in brightness is achieved for both systems.

Any positioning adjustment of the ODI can now be done in a reproducible manner with the new dual screw push‐pull mechanism. A large increase in efficiency is realized over that of the previous single screw trial and error mechanism.

The addition of the optical crosshair markings on the previously clear acrylic exit window facilitates an effective starting point for realignment and replacement of the reticule. The reticule crosshair is aligned optically with the projection from the acrylic window crosshair. Then centering of the crosshair is tested optically followed by a fluoroscopic test to verify that the optical and fluoroscopic crosshair images match.^3^ Now only one attempt for the proper positioning of the reticule crosshair suffices. Previously this was often a multi‐iterative approach between optical alignment and fluoroscopic alignment. Now after optical alignment the QC becomes simply a fluoroscopic check of the radio‐opaque reticule position to verify that the *x*‐ray center coincides with the optical center and the collimator axis of rotation.

## CONCLUSION

A Varian Ximatron simulator has been improved with regards to utility and the reduction of downtime. The field light and ODI assembly have been combined into one housing together with the cooling fan which now runs continually to remove any heating problems from the lights. This design makes these components more accessible and their adjustment independent of other components in the simulator gantry head. The field light has been modified to include fiber optics and any light bulb replacement occurs at a fixed position within the housing, eliminating time‐consuming optical maneuvering. Increased brightness to both the field light and ODI has been achieved with a tilting mechanism that fixes the bulb into a shadow‐free position within the assembly. The continuously running cooling fan reduces the heat enough such that it disallows the previously occurring displacement of the mirror. This saves much time and frustration in repair. Premature failure of the fan is also avoided. The ODI adjustment mechanism has been converted from a trial and error device utilizing a single screw into a dual screw push‐pull dynamic allowing reproducibility. A permanent optical crosshair that overlaps optically with the reticule crosshair has been added to the clear acrylic sheet composing the exit window. This allows an efficient reticule replacement or adjustment. All of these improvements have significantly reduced downtime of repair, replacement, and repositioning. The frequency of QC after routine adjustments has been greatly diminished with the added confidence of the design.
